# The paradoxical effects of high involvement work practices on employees and service outcomes: a trichromatic perspective

**DOI:** 10.3389/fpsyg.2024.1338171

**Published:** 2024-03-19

**Authors:** Xiaoxi Yang, Alia Qadir, Bilal Shahid, Safdar Husain Tahir

**Affiliations:** ^1^School of Economics and Management, Southwest Petroleum University, Chengdu, China; ^2^Department of Management Sciences, Riphah International University, Faisalabad, Pakistan; ^3^Institute of Business Management Sciences, University of Agriculture, Faisalabad, Pakistan; ^4^Lyallpur Business School, Government College University, Faisalabad, Pakistan

**Keywords:** subjective well-being, high involvement work practices, workload, customer orientation, work–family conflict, supportive leadership

## Abstract

This research delves into the complex impact of High Involvement Work Practices (HIWPs) on various facets of employee well-being and service outcomes within the framework of the trichromatic service conception. Utilizing the Job Demands-Resources (JD-R) model, the study uncovers the dual, both beneficial and detrimental, effects of HIWPs on service performance, work–family conflict, subjective well-being, and work-family enrichment. Examining the conflicting paths of job demands (workload) and job resources (customer orientation), the analysis incorporates the moderating influence of a strategic contextual factor—supervisor support. Data was collected through self-administered questionnaires from 475 respondents in Pakistani banks, and the analysis employed moderated mediation analysis using SPSS, AMOS, and the PROCESS Macro. All proposed hypotheses received support. The results indicate that HIWPs enhance service performance by promoting customer orientation but concurrently escalate workload, leading to adverse consequences for subjective well-being and work–family conflict. The study underscores the importance of implementing HIWPs under supportive leadership to maximize positive outcomes and mitigate negative consequences. Ultimately, this approach enables employees to effectively serve customers, maintain a healthy work-family balance, and contribute to the long-term growth and sustainability of organizations.

## Introduction

In today’s modern and highly competitive world, the service industry has emerged as a powerful element behind the global economy (Wang et al., 2022). Consequently, organizations recognize the pivotal role of employees as valuable assets (Wattoo et al., 2020; Collins, 2021). Effective management of employees directly impacts organizational efficiency, product quality, effectiveness, and profitability. To improve employee performance, researchers have extensively explored various factors (Bowen and Schneider, 2022) and shown particular interest in inclusive practices and processes that contribute to organizational success. However, researchers have disagreed on a singular approach to interpreting and operationalizing employee involvement (Litwin, 2015). High-involvement work practices (HIWPs) aim to establish more functional organizations by enhancing employees’ skills, motivation, and opportunities for participation, ultimately driving both employee and firm performance (Kim et al., 2021; Kaushik and Mukherjee, 2022). HIWPs are a set of exclusive but cooperative HR practices aimed at launching a better progressive organization (Ogbonnaya and Messersmith, 2019). These signify a “set” of mutually fortifying, overlying, and interdependent HR practices that highlight power, information, rewards, and knowledge, which provides assistance for participation and commitment of an employee” (Lawler, 1986; Lawler and Ledford, 1992; MacDuffie, 1995).

There are so many gaps pointed out by the researchers over time. Firstly, studies often focus on either positive or negative impacts, overlooking two contrasting perspectives: the optimistic view (mutual gains) and the pessimistic view (conflicting outcomes; Islam et al., 2023). Studies on positive relationships between HIWPs and employee outcomes are common, suggesting that employers and employees benefit from HIWPs, leading to positive employee behavior and improved organizational performance (Dongsen, 2023). However, research on potential harmful influences is rare (Han et al., 2023; Xiong, 2023), indicating either no effect or negative consequences on employees’ well-being (Bai et al., 2023). For balancing perspectives, additional research is required to assess HIWPs’ effects comprehensively with respect to organizational performance and their impact on the employee’s personal and family domains (Han et al., 2023).

Secondly, research in Western countries on HIWPs and employee outcomes is extensive, but there is a need for more research in Asian countries like Pakistan (Ahmad et al., 2020; Wang et al., 2022; Zhou et al., 2023) in order to determine the applicability and adaptability of these practices across different cultures and organizational perspectives. Cultural influences, workplace practices and organizational dynamics, may vary significantly across diverse counties and societies. Organizational objectives and employee well-being may have different connotations and implications. Family roles and work responsibilities are often deeply intertwined in these cultures. The existing research mainly mirrors Western organizational settings and perspectives. HIWPs effectiveness and applicability can vary in different settings because of the differences in employee expectations, leadership styles, and cultural norms. Understanding factors, boundary conditions, and job resources in these contexts is vital for maximizing HIWPs’ benefits and minimizing adverse effects.

Thirdly, identifying job resources or boundary conditions and parameters like supervisory support is crucial to coping with workplace intensification and job demands (Wattoo et al., 2020; Kaushik and Mukherjee, 2022). Studies emphasize the need for a framework incorporating HIWPs and the supportive role of supervisors to comprehensively understand their impact on employee well-being (Hauff et al., 2020; Bai et al., 2023). Organizational resources, such as supervisor support (SS), can effectively empower employees to handle work overload and associated job demands (Han et al., 2023). Research in this area, as highlighted by Kaushik and Mukherjee (2022), is essential for organizations to leverage strengths and address weaknesses through HIWPs implementation (Wang et al., 2022). Moreover, for a balanced and effective work environment, understanding the influence of HIWPs on work–family conflict and enrichment in these settings can provide valuable insights. This research, using two contrasting trajectories, the Job Demands-Resources model (JD-R model), explores HIWPs’ positive and negative effects on service outcomes, integrating ‘job demands and job resources’ perspectives (Kloutsiniotis and Mihail, 2020; Mokhtar and Krishnan, 2023).

Fourthly, researchers highlight the interconnectedness of personal, work, and family domains, with potential positive or negative effects (Han et al., 2023). Excessive work demands deplete employees’ time and energy, leading to work–family conflict (WFC) because more job involvement constrains full engagement in family roles (Allen et al., 2023). Moreover, unfavorable outcomes include decreased well-being and job dissatisfaction (Carlson et al., 2019). Conversely, research shows that balanced involvement in work and family domains can be advantageous, leading to work-family enrichment (WFE) and improved performance in both domains (Greenhaus and Powell, 2006). Limited research, especially in third-world countries, explores the impact of HIWPs on the work-family interface (Aubouin-Bonnaventure et al., 2023).

Fourthly, HIWPs aim to develop organizational competencies and achieve set goals, but often lead to strict performance measures and increased work demands (Ehrnrooth and Björkman, 2012). This results in employee distress due to heavy workloads and the continuous need for skill improvement, leading to physical, emotional, and behavioral distress (Xiong, 2023). While organizational goals and employee concerns may sometimes align, they can also diverge, potentially impeding employees’ ability to pursue personal life ambitions or corporate objectives. The relationship between HIWPs and their impact on well-being, including stress, health, and happiness, is underexplored, especially in third-world countries like Pakistan (Kaushik and Mukherjee, 2022; Han et al., 2023; Palumbo, 2023). The Pakistan Banking Perspective (2022) emphasizes a value-driven organizational culture, allowing employees to make informed decisions.

Fifthly, in this research, HIWPs aim to enhance skills, motivation, information sharing, and empowerment, measured using the PIRK framework proposed by Lawler (1986). These practices are expected to contribute to top-notch customer service (Liao et al., 2009), influencing employees’ service performance (SP) and subjective well-being (SWB). Existing literature often focuses on HIWPs’ positive effects, neglecting potential trade-offs employees may face in serving themselves or their families (Rubio-Andrés et al., 2022). This study aims to comprehensively evaluate the effectiveness of HIWPs by simultaneously addressing both positive and negative aspects.

Organizations can better understand how HIWPs affect service delivery by using a customer-oriented (CO) approach. It is necessary to conduct research to better understand the complex relationship between HIWPs and their impact on the delivery of services. Due to the significance of the banking sector in Pakistan’s economy, this study focuses on this sector (Chung et al., 2019). In order to improve HR management effectiveness, Pakistani banks have taken a number of initiatives to integrate HIWPs into their long-term strategic management frameworks (Hong et al., 2017). In light of these findings, researchers are urging further study of the fundamental mechanisms associated with these practices, which reaffirms their significance.

HIWPs have a dual impact, generating both work demands and work resources (Han et al., 2020). This study, using the Job Demands-Resources (JD-R) model, explores the conflicting effects of HIWPs on service outcomes and examines alternative job resources as potential buffers in the service industry (Han et al., 2023). For a number of reasons, authors examine the consequences within the context of the trichromatic service idea and explore the complex relationships that exist between workers, their clients, their families, and themselves. This study seeks to understand how frontline service employees simultaneously serve their clients (through CO), their families (via WFE and WFC; Li, 2023), and themselves (regarding SWB). The study adopts a two-fold approach, considering WL as a job demand and CO as a job resource, and examines the role of SS as a situational factor, which may amplify the positive effects of HIWPs through CO and mitigate the negative impacts through WL. Moreover, to fill the mentioned gaps, this study evaluates the positive and negative effects of HIWPs on Pakistani banking employee outcomes. Doing this will advance research on cultural and contextual aspects affecting HIWP effectiveness, a neglected field. Theoretically, this will enrich HIWP discourse using the JD-R model and PIRK framework. This unique method will systematically examine how HIWPs affect job demands, resources, service delivery, employee well-being, and work-family interactions. HR experts and organizational leaders in emerging nations and areas like banking where HIWP integration is still evolving can use these insights. This research may help them understand whether HIWPs are useful or harmful by giving a roadmap for designing and executing them to maximize their advantages and minimize their drawbacks by exploring even the effect of just a single element, like supervisory support, that will buffer HIWPs, work pressures, and employee well-being.

## Literature review and hypotheses development

### Theoretical framework

The JD-R model is widely recognized as one of the field’s most extensively researched and validated empirical frameworks (Lesener et al., 2019). This model proposes two fundamental pathways through which it operates: the straining and motivating processes. The straining process, also known as the destructive well-being pathway, suggests that long-term job demands or poorly designed work environments can lead to stress, deplete an employee’s psychological and physical resources, and negatively impact their SWB (Wang et al., 2022). In contrast, the motivational process posits that the accessibility of job resources within organizations, influenced by intrinsic and/or extrinsic factors, alleviates job pressures, facilitates goal achievement, fosters personal growth, and enhances SS.

This research focuses on HIWPs and their dual impact (i.e.) simultaneously providing job resources, such as service-oriented capabilities, ethics, and values, while also imposing job demands that can negatively affect employees’ SWB to enhance their skills and knowledge (Han et al., 2023) and JD-R model is uniquely positioned model to analyze this duality as it distinguishes between job demands and resources, providing a structured approach to assess how HIWPs can both strain and motivate employees. Further, this distinction can enhance the reader’s understanding of what type of effect HIWPs will have on employee well-being and performance (like increased responsibilities and skill development opportunities), which can either contribute to stress and resource depletion or foster employee engagement and resource accumulation. Moreover, this study also investigates the spillover effects of HIWPs on the work-family interface. The JD-R model has the capability to explain how job demands and resources influence work–family conflict and enrichment and delineates the pathways through which HIWPs impact employees’ personal lives, thereby offering a holistic view of the work-life balance. Besides, this study’s focus is Pakistan’s banking sector, a region-specific context. JD-R model is chosen here as it has flexibility and proven applicability across various cultural and organizational settings and has the capability to gauge the unique characteristics of Eastern workplaces and their impact on employee outcomes.

HIWPs encompass various inclusive practices, such as information exchange, training, and empowerment, that aim to enhance employees’ knowledge, abilities, and skills and grant them access to diverse social and economic resources (Jiang et al., 2012). However, the primary objective of HIWPs is to improve employee and organizational performance, which requires employees to invest greater commitment, time, and effort in skill development, resulting in increased responsibilities (Kroon et al., 2009). HIWPs can imbalance employees’ job needs and available work resources as claimed in the JD-R model. The resulting work pressures can deplete employees’ resources, leading to fatigue and stress (Boxall and Macky, 2014) and contributing to a heavy workload (Jensen et al., 2013).

The JD-R model also explains the spillover process, which describes how job resources and job demands influence the work-family interface, resulting in the employee’s family-related outcomes (Tement et al., 2023). The straining process suggests that high job demands may add to WFC, whereas the motivating process shows that job resources can lead to WFE. To enhance their performance and fulfill their responsibilities in their personal and work lives, employees often have to make sacrifices in terms of their time and energy, which can result in stress and challenges such as conflicts or emotional exhaustion between their job and family domains (Mariappanadar, 2014).

Considering the conservation of resources (COR) theory (Hobfoll, 2001), a dearth of resources frequently causes a decline in further resource loss, while preserving and/or gaining resources causes other resource gain. Moreover, when employees endeavor to achieve additional resources, they usually utilize the existing resources, creating “resource caravans” that support them in challenging times while helping them to thrive (Hobfoll, 2002). Further, it proposes that job resources act as the positive predictors of WFE, inferring that when employees have adequate job resources, it adds to a fulfilling and positive situation in their personal lives (Hakanen et al., 2011).

**Figure 1:**
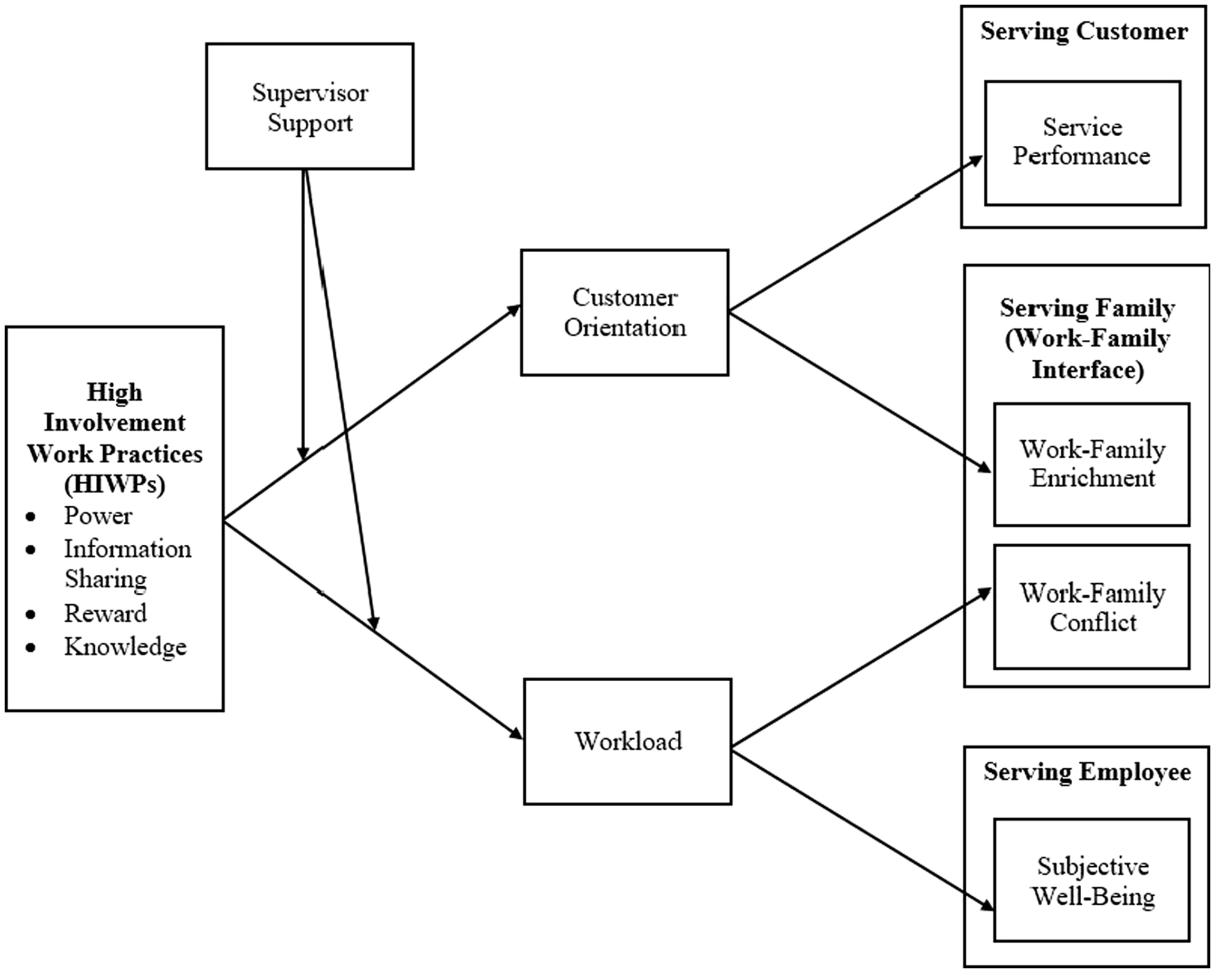
Conceptual framework.

Further, the JD-R model proposes that employee outcomes are affected by the integrated effect of job resources and demands (Bakker and Demerouti, 2007). The model’s safeguarding hypothesis further states that job resources can act as protective factors against the adverse effect of job demands on job stress (Bakker et al., 2003). Therefore, the COR theory and JD-R model argue that high work demands can deplete employees’ resources, leading to increased stress levels and negative behavioral and social outcomes. However, if employees can access work resources that help them cope with stress and overcome obstacles, they can better manage work demands. Both the JD-R model and the COR theory propose that the interplay between work resources and demands influences various outcomes examined in this study, including employee performance, the work-family interface and SWB. Figure 1 depicts the theoretical framework of the study.

### Development of hypotheses

In a service context employee’s customer orientation is a vital job resource and a guarantee of organizational success. It is a service value-system which draw the degree to which an employee’s work acuities, approaches, and actions are directed by a continuing credence in the significance of customer satisfaction (Zablah et al., 2012). According to Sousa et al. (2023), CO is a crucial job resource enabling employees to achieve service objectives, manage potential conflicts, and grow as service professionals (Park and Hur, 2023). These functions align with the PIRK model (measuring HIWPs) that predicted characteristics of job resources (Demerouti et al., 2001). Employee customer service and customer-centered perceptions, approaches, behaviors, and practices, encouraged and rewarded by the organizations, are enhanced and emphasized by HIWPs (Ali et al., 2022). Firstly, service-centered information-sharing practices emphasize the importance of planning and communicating customer-oriented strategies to employees. Secondly, financial and non-financial rewards are associated with employees’ SS through rewards-related practices. Thirdly, service-related training focuses on customer-centered approaches and actions, preparing employees to be attentive to customers’ needs and desires (Conduit and Mavondo, 2001). HIWPs may empower employees by granting them the authority to handle customer complaints in ways they deem appropriate, thereby enabling them to provide better customer service and potentially increasing their level of CO (Chan and Lam, 2011). Hence, it is proposed:

*H_1_: HIWPs have a positive association with employees’ CO.*

However, HIWPs also impose job demands on employees, which the employees can perceive as an increased workload (Ogbonnaya and Messersmith, 2019). To make employees high performers, training practices are provided to enhance their service skills, which are often provided during their regular working schedules, which can be burdensome for employees (Oppenauer and Van De Voorde, 2018). Additionally, performance evaluations and reward-related procedures usually emphasize the importance of service quality. This requires employees to work harder to achieve higher performance ratings, increasing job stress and burden (Palumbo, 2023). So,

*H_2_: HIWPs have a positive association with employees’ workload.*

Besides, customer-focused employees invest their time and effort in understanding and fulfilling the customers’ perceptions and needs, resulting in exceptional service quality (Aryee et al., 2019). They strive to maintain long-term relationships with customers, employ innovative sales techniques, pay close attention to customer preferences, and resolve any issues that may arise (Franke and Park, 2006). Knowing customer needs, participation, and involvement allows employees to make decisions and recommend refinements in service procedures but also necessitates an extra mental effort (Ogbonnaya and Messersmith, 2019). HIWPs incorporate training programs to help employees enhance their customer service-focused skills, knowledge, and abilities, while performance evaluations and rewards highlight employees’ commitment to delivering outstanding SP (Hong et al., 2013). By fostering a customer-focused workforce that prioritizes customer satisfaction, HIWPs are expected to impact SP positively (Aryee et al., 2019). Therefore,

*H_3_: Through the mediating effect of CO, HIWPs have a positive indirect impact on employees’ SP.*

HIWPs also impact how employees fulfill their responsibilities toward their families, as reflected in WFE and WFC, which are specifically related to job demands and job resources by representing two distinct aspects of the work-family interface. Particularly, WFE is expected to be developed by generating job resources via the motivating process, with CO as an intermediary factor (Tement et al., 2023). Based on the JD-R model (Bakker et al., 2011), previous research has shown that when employees have access to greater empowerment, enhanced skills, and even financial resources that can be utilized in their family domain, they are more likely to effectively manage their obligations at both work and home, resulting in positive work-family spillover (Wattoo et al., 2020). For nurturing HIWPs, the training programs that foster service attitudes may facilitate a positive spillover from the workplace to the home (i.e.) supporting employees in managing family-related issues and cultivating positive family behaviors (Han et al., 2023). Hence, it is proposed:

*H_4_: HIWPs have a positive indirect impact on employees’ WFE through the mediating effect of CO.*

Research has shown that WL drains employees’ resources and makes it difficult to allocate these effectively to family responsibilities, leading to higher levels of WFC (Michel et al., 2011). Following the straining process outlined in the JD-R model, WFC develops due to resource depletion and a straining process imposed by work tasks (Tement et al., 2023). HIWPs can potentially contribute to WFC through WL (Aubouin-Bonnaventure et al., 2023), making it difficult for employees to fulfill their family obligations and increasing their stress levels (Kim et al., 2021). Employees may feel compelled to extend their working hours, expedite service delivery, or exceed expectations to accommodate unpredictable client requests. This conflict between work and family domains arises due to the psychological, emotional, and physical energy expended at work, leaving fewer resources available for fulfilling family responsibilities (Kim et al., 2021). So, the researchers assume that,

*H_5_: HIWPs have a positive indirect impact on employees’ WFC, through the mechanism of WL.*

Furthermore, a higher workload can deplete employees’ emotional, cognitive, and physical resources, negatively affecting their physical and psychological health (Bowling et al., 2015). Although, HIWPs may promote employee empowerment, self-sufficiency, involvement, and decision-making, yet all these also introduce new responsibilities, requiring additional employee effort (Ramsay et al., 2000). Moreover, extensive training programs can enhance work complexity, elaborate performance evaluations, and demanding reward systems, which may lead to heightened job pressure and anxiety and culminate in negatively impacting employees’ SWB (Han et al., 2023). So, HIWPs are expected to positively associate with WL, a job demand that can potentially harm employees’ SWB. Therefore, it is hypothesized that:

*H_6_: HIWPs have a negative indirect impact on employees’ SWB through the mechanism of WL.*

According to the social exchange theory, employees who perceive their supervisors as supportive and helpful are more likely to increase their internal resources and improve customer-oriented behavior and service performance. So, the role of supervisors is crucial in guiding and motivating their employees as well as serving as role models in effectively meeting customers’ needs (Liaw et al., 2010) and enhancing employees’ customer-oriented behavior (Karatepe et al., 2007). Therefore, it is hypothesized that:

*H_7_: The indirect effect of HIWPs on employees’ service performance through CO will be strengthened when SS increases.*

Organizational resources are vital in facilitating personal development, learning, progress, and advancement across various aspects of an individual’s life, including physical, social, emotional, and work-related dimensions (Hakanen et al., 2006). According to the COR theory, individuals are motivated to acquire and protect resources as they recognize the value of resources in overcoming challenges. By having supportive supervisors, a perception is developed in the employees that their treatment at work is fair. Hence, their self-esteem increases, leading to higher motivation, commitment, and performance at work and in their family roles (Liao et al., 2016). The supervisor’s support can help employees balance their work and home responsibilities (Marais et al., 2014). Hence the researcher expects that:

*H_8_: SS will positively moderate the positive indirect relationship between HIWPs and WFE through CO.*

The COR theory posits that individuals experience stress when they perceive a scarcity or depletion of resources and strive to attain and preserve them (Hobfoll, 2001). Job intensification, characterized by increased workload and demands, can deplete an employee’s resources and negatively impact their performance in family roles. This leads to daily WFC, where individuals allocate more time and energy to work while neglecting family obligations (Boyar et al., 2024). SS is considered a valuable resource that helps employees effectively manage responsibilities in both work and family domains, promoting a sense of balance (Litano et al., 2016). SS facilitates employees in meeting work expectations and devoting sufficient time and effort to their families, thereby reducing WFC and alleviating job stress (Di Milia and Jiang, 2024). So, it is proposed that:

*H_9_: The positive indirect relationship between employees’ HIWPs and WFC through WL will be negatively moderated by SS.*

As previously mentioned, implementing HIWPs can lead to a straining process and increased job demands, potentially making work more complex and intense for employees (Kroon et al., 2009). This, in turn, depletes their valuable resources and negatively affects their well-being. Employees may experience decreased life satisfaction, hopelessness, emotional exhaustion, and physical strain (Kossek et al., 2024). SS is vital in helping workers manage the increased workload, reducing stress levels, enhancing well-being, and improving performance (Zhang and Song, 2020). Supportive managers prioritize their employees’ interests and professional development, creating a sense of recognition, belongingness, and inclusion. This helps employees mitigate the harmful effects of work overload and stress and significantly enhances their SWB (Han et al., 2023). On the contrary, a lack of supportive supervision deprives employees of the resources necessary to replenish their drained energy caused by HIWPs (Han et al., 2023). This leads to higher stress levels and, subsequently, poorer SWB. Therefore,

*H_10_: SS negatively moderated the negative indirect relationship between employees’ HIWPs and SWB through WL.*

### Data and methodology

This quantitative study focused on private banks in Faisalabad, as the banking industry is a significant contributor to Pakistan’s economy. This industry is characterized by its dynamic work environments and competitive pressures. It reflects Pakistani collectivistic culture that influences organizational dynamics, so it is ideal for studying how HIWPs interact with cultural values in a non-Western setting. The claim reflects that the collectivist nature of Pakistan is substantiated by the pervasive influence of collectivistic cultural values in organizational dynamics. Pakistani culture emphasizes strong interpersonal relationships, group harmony, and collective well-being over individual pursuits. In the banking sector, this manifests through organizational practices prioritizing group cohesion, loyalty, and employee mutual support. Teamwork and collaboration are highly valued, aligning with the collectivist ethos. This cultural backdrop, integral to Pakistan’s societal fabric, significantly shapes how HIWPs are adopted and impact organizational processes within the non-Western setting of Faisalabad.

The study employs a predictive, non-experimental survey design. The research adopted a cross-sectional approach, collecting data through a self-administered online questionnaire. Nonetheless, the questionnaire has received approval from the board of study, specifically the Department of Management Sciences at Riphah International University Islamabad, during its 13th meeting held on 6 July 2021. This research is conducted in Pakistan, which has a collectivistic culture that plays a pivotal role in shaping organizational dynamics and implementing HIWPs. Its norms and values about the organization, family and own self are quite different from those of the advanced countries with an individualistic culture. Pakistan, like many other Asian countries, is known for its collectivistic cultural values, emphasizing strong interpersonal relationships, group harmony, and collective well-being over individual pursuits. Organizations often prioritize group cohesion, loyalty, and mutual support among employees. Teamwork and collaboration are highly valued, and consensus-building may influence decisions rather than individual autonomy. This cultural backdrop has implications for the adoption and impact of HIWPs. The study seeks to unravel the intricacies of these connections within a cultural setting that differs significantly from the individualistic cultures often studied in advanced countries.

To gather responses, the researchers collaborated with the HR departments of selected private banks, sharing the URL link of the Google form with employees via WhatsApp and e-mail during personal visits to the bank offices. Clarification regarding the distribution process is vital for assessing any biases in the sample and understanding the influence of HR involvement in the data collection. Participants were identified, approached and assured that their information would be used only for study purposes and that their anonymity would be preserved. Hence, data was collected without identifying information such as names, employee IDs, or specific bank branches; responses were aggregated and analyzed at the group level to prevent individual identification. The online survey platform was configured to not collect IP addresses or any other tracking information. These measures were communicated to participants to encourage participation while maintaining anonymity and confidentiality. Data Collection took four and a half months. This study examined private Pakistani banks with more than 450 branches out of which at least 10 branches in Faisalabad spread across different areas, with a minimum of 20 years of existence, a total asset base of approximately PKR 500 billion (approx.), and a deposit base of approximately PKR 600 billion (approx.), having a market share of at least 5%, offering a comprehensive range of retail services, like savings accounts, loans, credit cards, etc., demonstrating consistent profitability and positive growth over the last three fiscal years, with a mix of recently established institutions (within the last 5 years). However, this criterion neglects smaller banks’ unique insights and practices. Focusing on banks with constant profitability and growth risks missing out on variations in HIWP implementation and outcomes across banking services. While covering banks of different eras offers diversity, it may not fully depict the evolving nature of HIWPs or long-term banking sector dynamics, particularly in Faisalabad’s private banks. Private banks are ideal for examining HIWPs’ organizational effects. Due to their decision-making autonomy, they can investigate more deeply without bureaucratic intervention than public banks. The flexibility of private banks’ HR policy shows how HIWPs adjust to organizational environments and market needs. Private banks’ willingness to adopt innovative HR practices makes them suitable for studying cutting-edge HIWP adoption and employee well-being and service outcomes. Private banks can respond quickly to market changes, allowing researchers to explore how HIWPs assist firms in overcoming challenges in a dynamic economy. Nonetheless, the questionnaire received approval from the board of study, specifically the Department of Management Sciences at Riphah International University Islamabad, during its 13th meeting held on July 6, 2021.

To ensure an appropriate sample size for SEM analysis, it was determined that the sample size must be more than five times the total number of questionnaire items (O’Rourke and Hatcher, 2013), which were 64 for this study. Consequently, the researchers initially aimed for a sample size of 320. However, to fulfill the SEM requirement for a sufficient sample size, the researchers opted for a single-stage increase, raising the final sample size to 475 (Wolf et al., 2013). The researchers used the simple random sampling technique, as it ensures every individual has an equal chance of selection, thereby offering a genuine representation of the population. This method not only reduces potential bias but is also cost and time-efficient. Its straightforward nature ensures replicability, enhancing the study’s credibility and utility for future comparative research. After an initial screening, 470 out of 650 survey questionnaires were selected for further analysis. All the questionnaires with missing values and erroneous responses were discarded.

### Demographic profile

The sample of 475 employees comprised of 63.8% (303) males 36.2% (172) females, 34.5% (164) Single 52.2% (248) Married, 7.4% (35) Divorced 5.9% (28) Widowed, 11.2% (53) employees belonged to 25–35 years age group, 32.8% (156) belonged to 36–45 years group, 31.6% (150) belonged to 36–45 years group, 6.7% (32) belonged to 56 and above age group, 19.4% (164) have Bachelor’s degree, 60.0% (285) have Master’s degree, 17.5% (83) have MS/MPhil, and 5.9% (15) have PhD degree, 50.3% (239), Frontline Service Employee, 33.5% (159) Middle Level Managers, 16.2% (77) Lower Level Managers, 14.7% (70) respondents have less than 5 years’ work experience, 29.7% (141) have 5–10 years’ experience, 28.8% (137) have 11–15 years’ experience, 15.4% (73) have 16–20 years, and 11.4% (54) respondents have more than 20 years of professional experience, 5.5% (26) have 40–50 working hours, 53.7% (255) have 51–60 working hours, 40.8% (194) have more than 60 working hours, see Table 1.

**Table 1:** Demographic profile of the respondents.

Demographic profile	Classification	*N* = 475	Percentage
Gender	Male	303	63.8
	Female	172	36.2
Age (in years)	Below 25	53	11.2
	25–35	156	32.8
	36–45	150	31.6
	46–55	84	17.7
	56 and above	32	6.7
Marital status	Single	164	34.5
	Married	248	52.2
	Divorced	35	7.4
	Widow/Widower	28	5.9
	Bachelors	92	19.4
Qualification	Masters	285	60.0
	MS/M.Phil.	83	17.5
	Ph.D.	15	3.2
Rank/position	Front Line Service Employee	239	50.3
	Lower-Level Manager	159	33.5
	Middle Level Manager	77	16.2
Professional experience	Less than 5	70	14.7
	5–10	141	29.7
	11–15	137	28.8
	16–20	73	15.4
	More than 20	54	11.4
Weekly working hours	40–50 h	26	5.5
	51–60 h	255	53.7
	More than 60 h	194	40.8

### Measurements

The assessment of 64 elements in the survey was conducted using validated and reliable instruments, see Annexure 1. The Likert scale, consisting of five possible outcomes that ranged from “strongly disagree (1) to strongly agree (5),” was used to assess the respondents’ attitudes and opinions. Mostly items were adopted, while others were slightly adapted to fit the context of the Pakistani banking sector.

Riordan et al. (2005) developed the PIRK model, which encompasses four standard practices: Power (3 items), Information Sharing (6 items), Reward (5 items) and Knowledge (4 items). In the study, these practices were used to evaluate HIWPs. A five-item scale developed by Bettencourt and Brown (1997) was used to measure Service Performance, while a nine-item scale developed by Carlson et al. (2019) had three dimensions: developmental, emotional, and capital-based work-family enrichment. Work-family enrichment based on development includes three items, work-family enrichment based on emotions includes three items and work-family enrichment based on capital includes three items. For measuring work–family conflict, a five-item scale by Netemeyer et al. (2005), subjective well-being a five-item ‘Satisfaction with Life Scale’ by Diener and Fujita (1995), and Customer Orientation, a five-item scale by Susskind et al. (2003), workload, a six-item scale by Harris and Bladen (1994) and Supervisor Support, a four-item scale by Karatepe and Olugbade (2009) were used.

The study utilized several measures to assess different constructs, as shown in Table 2. An online survey was conducted using Google Forms to collect data and obtain real-time responses. Participants were assured of their participation’s confidentiality, ensuring their responses’ privacy and anonymity. Only carefully completed and fully filled questionnaires were included. The collected data were coded and entered into an SPSS (Statistical Package for the Social Sciences) spreadsheet. Following data entry, multivariate assumptions were assessed to ensure the validity of subsequent analyses. CB-SEM instead of PLS-SEM was chosen due to its suitability for theory testing and confirmation, as this study focuses on validating existing theoretical frameworks in a new context and also due to the nature of its objectives and the characteristics of the data. Moreover, the sample size and the scale of measurement models were adequate for CB-SEM having rigorous model fit assessment capabilities. All four assumptions of SEM were met in this study. The normality of the data was assessed by examining kurtosis and skewness values, which were within the acceptable range, pointing to a normal distribution of the data.

**Table 2:** Summary of measurement scales.

Variable	Code	Author	Items
High Involvement Work Practices (HIWPs)	HIWPs	Riordan et al. (2005)	18
Service Performance	SP	Bettencourt and Brown (1997)	5
Work-Family Enrichment	WFE	Carlson et al. (2006)	9
Work–Family Conflict	WFC	Netemeyer et al. (2005)	5
Subjective Well-Being	SWB	Diener and Fujita (1995)	5
Customer Orientation	CO	Susskind et al. (2003)	5
Workload	WL	Harris and Bladen (1994)	6
Supervisor Support	SS	Karatepe and Olugbade (2009)	4

To address the concern of common method bias (CMB), a methodological framework (Harman One Factor Test) suggested by Henseler et al. (2015) was adopted. The universally accepted threshold for common variance is below 50%. This criterion serves as a reliable indicator, suggesting that when the common variance is less than 50%, there are no concerns related to CMB (Henseler et al., 2015). The outcomes of the Harman One Factor Test reveal a common variance of 46.079% for the dataset. This value falls below the established threshold of 50%. For a comprehensive breakdown of the Harman One Factor Test results pertaining to CMB, refer to the detailed findings in Annexure 2. The results from this analysis indicates that CMB is not a predominant issue.

Multicollinearity was evaluated using Variance Inflation Factor (VIF), and all VIF values were below 3, ranging between 1.5 to 2.3, indicating that multicollinearity was not an issue. Data analysis was performed using the SEM approach with the AMOS software. First-order confirmatory factor analysis (CFA) and second-order CFA were conducted in accordance with the recommendations by Anderson and Gerbing (1988).

A specification search for CFA involved 70 first-order latent variables and 57 observable variables. Maximum likelihood estimation (MLE) was employed for model assessment. To evaluate model fit, first-order factor analysis was conducted, which provided factor loadings, AVE, SMC range, and Cronbach’s alpha (α) values. The measurement model results demonstrated that α scores of the measures ranged from 0.905 to 0.982, indicating satisfactory reliability (as shown in Table 3). Moreover, composite reliability (CR) values exceeded the acceptable threshold, ranging from 0.934 to 0.974 (see Table 4). The AVE measurement for all variables ranged from 0.752 to 0.905, surpassing the minimum criterion of 0.50 (as shown in Table 5), confirming convergent validity. Furthermore, the substantial factor loadings of the measurement items provided additional evidence for convergent validity.

**Table 3:** Study constructs’ Cronbach’s alpha (α).

Sr. No.	Constructs	Dimensions	Cronbach’s alpha (α)
1	*Service Performance (SP)*		*0.941*
2	*High Involvement Work Practices (HIWPs)*		*0.982*
		HIWPs-Reward	0.938
		HIWPs-Information Sharing	0.955
		HIWPs-Knowledge	0.938
		HIWPs-Power	0.905
3	*Work–Family Conflict (WFC)*		*0.950*
4	*Work-Family Enrichment (WFE)*		*0.963*
		WFE-Capital	0.920
		WFE-Affect	0.910
		WFE-Development	0.917
5	*Customer Orientation (CO)*		*0.952*
6	*Subjective Well-being (SWB)*		*0.938*
7	*Supervisor Support (SS)*		*0.931*
8	*Workload (WL)*		*0.952*

**Table 4:** Composite reliability and convergent validity.

Construct	CR	AVE	MSV
HIWPs	0.974	0.905	0.627
SP	0.943	0.770	0.656
WFE	0.949	0.863	0.659
WFC	0.951	0.794	0.658
SWB	0.938	0.752	0.696
CO	0.953	0.801	0.659
WL	0.953	0.772	0.696
SS	0.934	0.780	0.604

**Table 5:** Discriminant validity.

Construct	SWB	HIWPs	CO	SP	WFE	WL	WFC	SS
SWB	**0.867**							
HIWPs	−0.315	**0.951**						
CO	−0.599	0.680	**0.895**					
SP	−0.727	0.792	0.791	**0.878**				
WFE	−0.615	0.691	0.812	0.810	**0.929**			
WL	−0.834	0.603	0.618	0.627	0.610	**0.879**		
WFC	−0.811	0.564	0.572	0.583	0.567	0.767	**0.891**	
SS	0.777	0.577	0.605	0.566	0.474	−0.475	−0.583	**0.883**

Bold values in the Table 5 are Square roots of AVE.

Six constructs, namely SWB, CO, SP, WFC, WL, and SS, were considered first-order constructs during the CFA. Additionally, WFE and HIWPs were treated as second-order or higher-order reflective constructs. Reliability, discriminant, and convergent validity were assessed while examining the measurement model. All factor loadings were significant, so convergent validity was proven.

To establish discriminant validity, the squared inter-construct correlation coefficients were compared with AVE square root. The results confirmed the presence of discriminant validity, as the correlation coefficients between constructs were all below the square root of AVE, indicating that they were significantly different from one another. Table 4 provides further details on these results.

However, the initial model statistics fell slightly below the suggested threshold, indicating that re-specification of the measurement model was necessary to achieve an excellent fit. The decision to re-specify the model was made to improve the overall model fit, as shown in Table 6. Moreover, Table 6 presents the values of default model before modification (known as initial model) indices and the values of the model after modification (known as final model) indices during CFA. After modification indices are the values after all adjustments in the measurement and structural models.

**Table 6:** Initial and final measurement and structural models.

Fit indices	Initial measurement model	Final measurement model	Initial structural model	Final structural model	Ranges and acceptance criteria	Analysis of final measurement model
CMIN/df	3.694	2.306	3.751	2.382	<3 Good	Good fit
GFI	0.718	0.912	0.743	0.916	>0.95 Great	Good fit
AGFI	0.670	0.828	0.703	0.846	>0.80 Great	Good fit
CFI	0.936	0.967	0.941	0.950	>0.95 Great	Good fit
RMSEA	0.075	0.042	0.076	0.044	0.50 to 0.10 Mod.	Mod. fit

### Hypotheses testing

The next step was evaluating the structural model fit to examine the hypothesized relationships among all exogenous and endogenous variables. It was derived from Hayes’ theoretical/conceptual model, consisting of eight variables and 15 indicators. Within the model, one variable (SS) served as a moderator, two variables (CO and WL) functioned as mediators, one variable (HIWPs) acted as an exogenous variable, and four variables (WFC, WFE, SP, SWB) were considered endogenous variables. All model fit indices in Table 6 exceeded the acceptable lower-limit values presented by Hu and Bentler (1999), indicating a good fit of the structural model to the data. No paths needed to be eliminated from the model. The model demonstrated a satisfactory level of fit according to the specified threshold criteria.

#### Direct effect analysis

From Table 7, the relationship between HIWPs and CO was highly significant (0.638, *p* < 0.05), supporting H1. Similarly, the results indicated that HIWPs accounted for 58.6% of the variance in WL (0.586, *p* < 0.05). This significant and positive association between HIWPs and WL further supported H2.

**Table 7:** The standardized direct effect.

Hypotheses	Structural paths	Standard regression coefficient	Sig.	Result
H_1_	HIWPs → CO	0.638	***	Accepted
H_2_	HIWPs → WL	0.586	***	Accepted

The symbol ***indicates that the level of significance at 1%.

#### Mediation analysis

A bootstrapping method was employed using 5,000 bootstrap samples and a 95% bias-corrected confidence interval to assess total, direct, and indirect effects. Mediation analysis (shown in Table 8) was conducted in AMOS-24, which allowed for simultaneous evaluation of these effects. With a two-tailed test, the bootstrapping significance value provided information on the significance levels of the direct, indirect, and total effects. According to the study, HIWPs accounted for 30.4% of SP variance directly related to HIWPs. However, when CO was added between HIWPs and SP, this influence increased to 45.2%. CO significantly mediated the relationship between HIWPs and SP, but the mediation was only partially due to the weakened direct relationship. However, a statistically significant relationship remained between these, as indicated by less than 0.05 significance value of the bootstrapping two-tailed test. These findings support the presence of partial mediation and highlight the importance of the indirect impact of CO, thus accepting H3.

**Table 8:** The standardized indirect, direct, and total effect.

Hypotheses/Paths	Direct effect		Indirect effect		Total effect		Mediation level
	Coeff.	Sig.	Coef.	Sig.	Coeff.	Sig.	
H_3_: HIWPs → CO → SP	0.304	***	0.452	***	0.756	***	Partial
H_4_: HIWPs → CO → WFE	0.224	***	0.438	***	0.662	***	Partial
H_5_: HIWPs → WL → WFC	0.170	***	0.368	***	0.538	***	Partial
H_6_: HIWPs → WL → SWB	−0.140	*	−0.216	***	−0.356	***	Partial

The symbol * and *** indicates that the level of significance at 10% and 1%, respectively.

The mediating factor between HIWPs and WFE was identified as CO, which was found to strongly but only partially mediate the relationship between the two. The indirect effect (43.8%) was considerably stronger than the direct effect (22.4%). The total effect showed a positive relationship between HIWPs and WFE (0.662; *p* < 0.05). So H4 was proved. According to H5, HIWPs had a statistically significant positive relationship with WFC (0.538, *p* < 0.05). When the mediating variable WL was included, the direct impact of HIWPs on WFC remained significant (0.170, *p* < 0.05), indicating that mediation existed in this relationship, and the indirect effect was 36.8%. As a result, H5 was accepted. The direct impact analysis showed that between HIWPs and SWB, a significant negative relationship existed (−0.356; *p* < 0.05). Even after including WL as a mediating variable, the direct effect remained significant (−0.140), while the indirect effect became more pronounced and significant (−0.216; *p* < 0.05). These results indicate that WL partially mediated the association between HIWPs and SWB. Therefore, H6 was also accepted.

#### Moderation mediation analyses

Moderated mediation analysis (as shown in Table 9) was conducted using the PROCESS Macro for SPSS version 3.4.1 (Hayes, 2015). The index of moderated mediation was generated by employing a bootstrapping method with 5,000 samples to obtain bias-corrected confidence intervals at a 95% level (Preacher et al., 2007). The results indicated that SS significantly strengthens the direct relationship between HIWPs and SP, as evidenced by both the interactions’ Z-Score and value of p being less than 0.05. The index of moderated mediation further confirmed this effect, with the lower-level confidence interval (LLCI) and upper-level confidence interval (ULCI) values reported as [0.0078, 0.0096], [0.0041, 0.0053], [−0.1635, −0.0565], and [0.0539, 0.1580] for SP, WFE, WFC, and SWB, respectively. These results demonstrate moderated differences in the conditional indirect effect when the bootstrapping two-tailed significance values were below 0.05. Therefore, it can be concluded that SS independently enhances the indirect impact of HIWPs on SP, WFE, WFC, and SWB through the mediating variables of CO, and WL. Consequently, H7, H8, H9, and H10 are accepted.

**Table 9:** The conditional indirect effect of moderated mediations.

*Process Macro: Model 7*				
*Moderated mediation analysis with supervisor support as moderating variable*				
**Hypotheses**	**Index**	**Boot SE**	**Boot LLCI**	**Boot ULCI**
H_7_: SS × HIWPs → CO → SP	0.0006	0.0044	0.0078	0.0096
H_8_: SS × HIWPs → CO → WFE	0.0003	0.0023	0.0041	0.0053
H_9_: SS × HIWPs → WL → WFC	−0.1124	0.0272	−0.1635	−0.0565
H_10_: SS × HIWPs → WL → SWB	0.1080	0.0266	0.0539	0.1580

## Discussion

This study is grounded in the JD-R model and the COR theory, providing insights into the effects of HIWPs on various outcomes. The key findings confirm the dual nature effect of HIWPs, by proposing two offsetting mechanisms of customer orientation and workload acting as mediators. HIWPs positively and significantly contribute to Service Performance by enhancing Customer Orientation while negatively impacting Subjective Well-being through Work Load. The perception of HIWPs regarding employee support for their families, as reflected in Customer Orientation and Work Load, exhibited variation. Furthermore, the results demonstrate that Supervisor Support, as a job resource, is a strong moderator, as evident from its amplifying the positive effects of HIWPs, such as Service Performance and Work-Family Enrichment, while attenuating the adverse effects, such as Work–Family Conflict and Subjective Well-being.

All the results confirmed the prior literature. It is evident coinciding with the results of Wang and Chang (2016), the results of H1 indicate that HIWPs in bank employees lead to increased customer satisfaction by enhancing their customer-centered insights and behaviors, due to their focus on skill enhancement, empowerment, and participatory decision-making. This alignment helps employees fulfill service goals and gives them the tools and mentality to manage disagreements and flourish as service professionals. Moreover, these findings align with the PIRK model suggesting that HIWPs are employment resources that help enhance service delivery skills. H2 findings reveal similar findings as those of Zahoor et al. (2021) that HIWPs also contribute to increased work-life conflict among employees due to the demands of working longer hours, delivering services quickly, and meeting client expectations. Since HIWPs strive to improve service quality and employee engagement, they also increase job expectations, which supports the hypothesis. Extensive training, performance assessments, and pressure to fulfill greater service standards can increase staff workload. H3 results demonstrate that HIWPs enhance employees’ CO, leading to improved performance and exceeding expectations in their roles, increasing their job satisfaction. The findings were consistent with those of Karatepe et al. (2018) and Lapierre et al. (2018). Moreover, customer-focused employees are more likely to understand and meet client needs, resulting in better service. HIWPs provide customer-focused training, incentives, and rewards, indirectly impacting SP through CO. H4 results indicate that HIWPs promote customer-focused behaviors, positively impacting employees’ work and family lives and generating psychological and emotional resources that help them effectively fulfill their family responsibilities. These results are consistent with prior research by Michel et al. (2011). CO’s mediating role shows that customer service personnel who feel supported are more likely to feel accomplished and satisfied, which benefits their family life. Findings of H5 propose that the widespread use of HIWPs strengthens employees’ work engagement, resulting in depleted resources for addressing family issues and consequently leading to higher WFC. These findings support the conclusions of Michel et al. (2011). H6 demonstrates that HIWPs indirectly negatively impact employees’ SWB through increased WFC. HIWPs impose additional responsibilities on employees, which leads to higher work expectations, longer working hours, increased anxiety, feelings of despair, fatigue, and physical symptoms that negatively affect employee SWB. These findings correspond to those of Bowling et al. (2015). Conclusively, HIWPs have positive and negative effects on employees, impacting customer satisfaction, work-life balance, job satisfaction, work engagement, and subjective well-being. The results highlight the importance of considering the potential benefits and drawbacks of HIWPs in the banking sector.

In line with the findings of Eren et al. (2013), H7 results demonstrate that employees who perceive Supervisor Support have additional internal resources that enable them to meet customer requirements and exhibit customer-oriented behavior, leading to enhanced customer satisfaction and Service Performance. H8 and H9 findings indicate that employees with supportive supervisors experience positive outcomes at work and home or vice versa. The findings coincide with COR theory and also with the prior studies by Karatepe et al. (2007), Marais et al. (2014), Goh et al. (2015), and Mansour and Tremblay (2016) demonstrating the vital role that support from organizations plays in achieving an effective integration of job and family duties. HIWPs are prone to work overload, which exhausts individuals’ energy, time, and emotional resources. WFC may result from an employee’s constant depletion of resources due to increased work demands; by empowering workers with the mental and practical resources needed to meet these demands and providing them with the flexibility needed to dedicate more time and energy to their families, supportive managers can mitigate the adverse effects of HIWPs like WFC. Employees engage in affectionate and responsive interactions with their families, promoting WFE. Moreover, supportive leadership reduces the stress that results from increased job demands. Employees working in supportive organizational environments with supportive managers can better meet customer needs, achieve a work-family balance, and manage the challenges posed by HIWPs, which is essential to the well-being of employees and the organization’s success.

Hypothesis 10 findings indicate that HIWPs may increase employees’ challenges and demands by potentially depleting their vital resources, leading to decreased life satisfaction, despondency, emotional exhaustion, and physical strain (Goh et al., 2015). These harmful effects can, however, be mitigated by the supervisor’s supportive behavior. Supervisors’ perception of inclusion, belonging, and acknowledgment can increase workers’ life satisfaction and subjective well-being (SWB). Consequently, supervisory support can act as a shield, minimizing the adverse effects of job stress and overload. Employees who feel valued and supported are less likely to experience the negative consequences of job tension and overload, leading to higher life satisfaction and job satisfaction.

Overall findings of this study unearth and prove many facts about different relationships and their theoretical background. Human nature, attitude, and behavior remain the same regardless of culture. It is collectivistic or individualistic; the only difference in the manifestation is its intensity, duration, and how it is expressed. Based on the JD-R model and COR theory, this study was conducted in a country with a collectivistic culture; the findings confirm the dual impact of HIWPs on employee outcomes, expand the literature in various ways, and provide unique insights into how HIWPs affect service performance and staff well-being in the banking sector, where supervisor support acting as a moderator reveals how managerial actions can maximize or minimize these effects. In Pakistan, where hierarchical and relational dynamics are prominent, the supportive role of supervisors is magnified and can significantly influence the effectiveness of HIWPs. It can lead to heightened employee loyalty and a strong sense of belonging, pivotal in collectivistic societies. Moreover, the findings provide empirical evidence of how customer orientation and workload as mediators can affect these mechanisms through which HIWPs influence positive and negative employee outcomes. Moreover, customer orientation enhances service performance through collective efforts and shared successes. However, it poses a complex situation as HIWPs are aimed at improving employee engagement and organizational performance, they also inadvertently increase workload, which can be particularly challenging in a collectivistic context where work-life boundaries are often blurred, and familial obligations are deeply ingrained. This can intensify work–family conflict, challenging the traditional support structures central to Pakistani society. This detailed knowledge clarifies the conditions under which HIWPs can be most effective and beneficial. Finally, the findings provide evidence-based, culturally appropriate guidelines for using HIWPs to improve service performance and work-family enrichment in ways that respect collectivism without exacerbating work–family conflict or undermining subjective well-being. So, the findings present a balanced HIWPs’ implementation approach, which gives organizations new ways to navigate the complexities of modern work practices while honoring traditional values of collectivism and family cohesiveness.

### Practical implications

This research has a number of practical implications for managers in Pakistan’s banking sector. This research focused not only on enriching the global academic discourse but also on helping to develop more contextually relevant management practices. First, in line with prior literature, the study findings demonstrate that HIWPs are an effective and advantageous management practice system for improving employee performance. Hence, to maintain service quality and profitability, private banks are supposed to implement HIWP practices. Although this study reveals the detrimental effects of HIWPs on the subjective well-being of employees as well as work–family conflict, banks must develop a more advanced vision of HIWPs to reach their objectives. The most crucial aspect of HIWP is to be cognizant of its effects on employees’ perception of added workload. There must be implementation of policies that promote work-life balance; that is, employees must be provided with stress management programs and policies to cope with job demands, along with work-family supportive plans and policies. As a means of resolving conflicts between work and family responsibilities (Butts et al., 2013), banks should provide flexible working hours, child care facilitation on-site, etc. In line with the results of Wang et al. (2022), the findings suggest that banks should promote the happiness, pleasure and life satisfaction of their employees by providing such programs and protecting them from the destructive effects that may result from the increased job demands caused by HIWPs.

Additionally, banks should enhance employees’ customer orientation, which enhances their service performance and enriches their work-family balance. Lastly, it is essential to note that it is not suggested by this research that HIWPs should not be implemented in service organizations when managing employees in the service sector. Besides, the study recommends that care should be done while applying HIWPs, the managers and organizations should also be aware of the possible costs associated with this initiative. So, the study’s empirical findings highlight the importance of managers demonstrating supportive leadership behavior to their employees when implementing their HIWPs. Organizations should invest in training programs that equip supervisors with the skills to provide emotional and instrumental support, recognize employee efforts, and foster a supportive team environment. Such training should also emphasize managing workloads and helping employees balance work and family demands. The behavior of Supervisor Support contributes to increasing the desirable effects of HR practices and decreasing their undesirable effects. Moreover, employees play a crucial role in service performance, as their work-family integration and personal life satisfaction are vital for the sustainability and profitability of an organization.

### Theoretical implications

This research makes substantial theoretical contributions by extending and enriching the understanding of COR and JD-R models in the context of HIWPs and their impact on employee outcomes. Firstly, by challenging the prevailing assumption that HIWPs uniformly yield positive outcomes, this study prompts a reevaluation of the existing theoretical paradigms. The dual perspective of HIWPs aligns with the COR theory, which posits that individuals strive to acquire, retain, and protect resources, thereby emphasizing the need to consider potential resource losses and gains associated with HIWPs. Moreover, this aligns with the foundational principles of both COR and the JD-R model, as the former underscores the importance of resource investment and conservation, while the latter explicates the dual influence of job demands and resources on employee outcomes. Secondly, in alignment with the JD-R model’s emphasis on the dual processes of job demands and resources influencing employee well-being and performance, our study extends the JD-R model’s application to encompass broader organizational dynamics by empirically demonstrating the impact of HIWPs on workers’ attitudes toward customers, coupled with insights into balancing work and family obligations.

Another contribution of the study is that it enhances our understanding of how HIWPs and WFB interact. HIWPs are empirically demonstrated to impact workers’ attitudes toward customers and family and demand that work and family obligations should be balanced. The study findings underscore the complexity of this dynamic by providing insight into the benefits and disadvantages of HIWPs by demonstrating the advantages and disadvantages they introduce to diverse employee outcomes via multiple processes. In addition, this complex awareness provides a basis for identifying contextual limitations and highlighting the need for proactive measures to enhance the positive effects of HIWPs while minimizing their adverse consequences.

## Conclusion

Based on the findings of this study, HIWPs do not represent an all-encompassing solution, nor are they inherently detrimental. As a result, the authors emphasize the importance of developing a comprehensive strategy that considers both the positive and negative aspects of HIWPs. Organizations should devise policies that can help employees perform their family and work obligations, so taking initiatives for stress management and helping employees achieve work-family balance has been recommended to minimize potential conflicts and improve employee well-being. Besides, it also emphasizes the importance of management support in maximizing the positive effects of HIWPs. In short, it suggests that HIWPs should not be rejected but rather be applied carefully, considering their potential costs and using those practices that promote employee satisfaction and well-being.

### Limitations and future recommendations

To enhance precision and clarity, future research may focus on a few key areas to overcome this study’s limitations. This study is limited by using self-reported instruments to measure nearly all variables, which may contribute to a common method bias. In order to mitigate this issue, it is suggested that future research diversify its data sources and ensure the anonymity of the respondents. This may involve incorporating objective performance indicators, peer and supervisor feedback, or direct observations. In addition, employees’ reluctance to share work-related information with their families may have affected the study’s findings. Further research may benefit from a longitudinal design to provide a more comprehensive understanding of employee outcomes.

This method would circumvent the limitations imposed by the cross-sectional nature of the current study. It should also be noted that the present research is situated within the collectivistic culture of Pakistan. Consequently, research findings cannot be similarly applied to other sectors or cultures. This study’s theoretical framework should be applied to various business and social settings in future studies. The study also demonstrates that banks priorities customer satisfaction over employee well-being and work-life balance. It is necessary to identify industry-specific patterns and cultural factors that may influence the outcomes of HIWPs in order to understand their impact. HIWPs can be better understood through studies across various industries and cultures. This study uses the JD-R paradigm to understand the complex implications of HIWPs; however, there is the possibility that other mechanisms may also contribute to the results. In order to enhance the positive effects of HIWPs and mitigate their negative effects, future research should investigate alternative mechanisms and additional limitations. Other resources, both personal and environmental, such as resilience, self-efficacy, work engagement and peer support, may need to be examined.

## Data availability statement

The original contributions presented in the study are included in the article/supplementary material, further inquiries can be directed to the corresponding authors.

## Author contributions

XY: Conceptualization, Formal analysis, Funding acquisition, Investigation, Methodology, Project administration, Resources, Software, Visualization, Writing – review & editing. AQ: Data curation, Investigation, Software, Visualization, Writing – review & editing, Supervision, Writing – original draft. BS: Conceptualization, Formal analysis, Methodology, Resources, Software, Writing – original draft, Data curation, Writing – review & editing. ST: Funding acquisition, Project administration, Supervision, Validation, Visualization, Conceptualization, Formal analysis, Writing – review & editing.
